# Sunflower seed allergy in a child reveloping after sensitization to sunflower pollen or dust

**DOI:** 10.1186/2045-7022-1-S1-P110

**Published:** 2011-08-12

**Authors:** Pinar Uysal, Zeynep Arikan Ayyildiz, Fatih Firinci, Tuba Tuncel, Dilek Tezcan, Ozkan Karaman, Nevin Uzuner

**Affiliations:** 1 Dokuz Eylul University Faculty of Medicine, Department of Pediatric Allergy, Izmir, Turkey

## Introduction

Sunflower seed is one of the most commonly consumed snacks in our country. Sunflower seeds are not only eaten as snacks but also are used as ingredients of breads, mueslies and bars in recent years. Sunflower seed allergy is seen rarely in children and may be presented as angioedema or anaphylaxis. Respiratory allergies have been reported previously in adults owning birds fed on sunflower seeds. We present a case with sunflower seed allergy who had been already sensitized with sunflower pollen or dust and developed sunflower seed allergy afterwards.

## Case report

A 9- year boy, with a history of allergic rhinoconjunctivitis and asthma reported that he experienced generalized urticaria and facial angioedema within minutes after consuming small amounts of sunflower seeds. He had similar episodes three times since the age of 3 years. He told that he experienced worsening of respiratory symptoms since infancy when he traveled to his homeland where sunflower harvest was held in summers. Allergy was confirmed by a positive prick to prick test to sunflower seeds, positive specific IgE (12.7 kU/l) (Phaida, Uppsala, Sweden), and a positive oral challenge test. After ingestion of 15 g of sunflower seed, he developed severe vomiting, urticaria and itchy, red eyes. His previous skin prick test was positive for grasses, cereals, pines, weeds, mugwort and animal dander. Specific Ig E for sunflower pollen was also found positive (11.1 kU/l) (Phaida, Uppsala, Sweden).

## Conclusion

Sunflower seed allergy is rarely seen in children. This is the first reported pediatric case who developed sunflower seed allergy after senstization with sunflower pollen or dust. We presented this case to also underline that sensitization by inhalation may also precede food allergy in the future.

